# Thermal and Mechanical Characterization of Banana Fiber Reinforced Composites for Its Application in Injection Molding

**DOI:** 10.3390/ma13163581

**Published:** 2020-08-13

**Authors:** Dragan Kusić, Uroš Božič, Mario Monzón, Rubén Paz, Pablo Bordón

**Affiliations:** 1 TECOS Slovenian Tool and Die Development Centre, Kidričeva 25, 3000 Celje, Slovenia; dragan.kusic@tecos.si; 2 Ambi-Metalplast d.o.o, Smolenja vas 11D, 8000 Novo Mesto, Slovenia; uros.bozic@ambi.si; 3 Mechanical Engineering Department, Universidad de Las Palmas de Gran Canaria, Edificio de Ingenierías, Campus de Tafira Baja, 35017 Las Palmas, Spain; ruben.paz@ulpgc.es (R.P.); pablo.bordon@ulpgc.es (P.B.)

**Keywords:** natural fiber composites, banana fiber, fiber reinforcements, extrusion and injection molding techniques, short fiber thermoplastics, thermoplastics matrix

## Abstract

Several natural materials and vegetable waste have relevant mechanical properties, mainly in its fiber format. Particularly, banana fiber (BF) provides a close behavior to the widely spread glass fibers, which places it in an advantageous position for use as a reinforcing material in plastic composites. This work characterizes the behavior of acrylonitrile butadiene styrene (ABS), high impact polystyrene (HIPS), and high density polyethylene (HDPE) reinforced with short fibers of bananas from the Canary Islands for its application in molding processes. Several thermal analyses (Thermal Gravimetric Analysis (TGA), Differential Scanning Calorimetry (DSC), and Melt Flow Index (MFI)) and mechanical tests (tensile, flexural, impact, and Dynamic Mechanical Analysis (DMA)) were carried out in composites with different percentages of banana fiber. The thermal results show that the use of banana fiber is viable as a reinforcement in composites for injection molding processes and the mechanical tests indicate an increase in stiffness and an improvement in maximum flexural stress by increasing the fiber content in composites, so the banana fiber turns out to be a natural alternative for the reinforcement of injected plastic components.

## 1. Introduction

Despite the current effort to promote circular economy, according to 2015 data [1], from 6300 Mt of plastic waste, only 9% was recycled, 12% incinerated, and 79% sent to landfills. Interest in the use of natural materials classified as waste or by-products of other processes continues to grow among the scientific community to address how to introduce them, for example, as additives for industrial plastic components, what effects they produce when incorporated and the possibilities of forming them through processes such as injection molding.

Several are the natural materials and vegetable waste that may have relevant mechanical properties, mainly in its fiber format, and which have been characterized by various authors in recent years. As observed in Table 1, showing a range of minimum and maximum values of mechanical data analyzed by different authors, the mechanical behavior of the different natural fibers is highly variable, significantly affecting the process of obtaining and pretreatment [2,3], in addition to the variability of the species itself, the specific conditions of the environment in which each plant develops, the moisture content or the uncertainty associated with the cross-section measurement process for calculating the stress–strain curve [4,5]. Classifying these fibers by their specific tensile strength, and except for the high performance of kenaf, the fibers obtained from the Canary banana are among the highest performance fibers, surpassing sisal, hemp or jute, and presenting a close behavior to the widely spread glass fibers, which places it in an advantageous position for use as a reinforcing material in plastic composites.

In the specific case of banana fiber in the Canary Islands, with an annual production of crop-derived residues of about 17 million pseudostems per year [11], the conversion of this residue into a natural resource (through the reuse of the banana pseudostem using an innovative technology for the extraction of natural fiber and pulp) can produce both an environmental benefit (derived from the improvement in the management of this agricultural waste) and the development of a circular economy in this sector. A study in the Canary Islands [11] concluded that the exploitation of approximately 60% of the total pseudostems generated (considering only the usable length), would represent an estimated production of 2000 t of dry fiber and 7000 t of dry pulp per year (pulp suitable for the production of additives for animal feed).

The use of natural fiber from banana crops continues to expand with a wide range of applications, such as the improvement of reinforced technical textile composite by compression molding [12], the use of short fiber for rotational molding composite [6], filters for water purification [13] or the production of micro/nano fibers for the paper making sector [14]. Short fibers can be more easily adapted to the most widespread plastic forming processes, such as injection molding, being therefore a suitable fiber configuration for the reinforcement of injected plastic components. However, this configuration also has some limitations: (a) the previous manufacturing of pellets of natural fiber composites (NFCs) by extrusion is not always efficient enough in terms of uniform distribution of fiber (matrix–fiber), (b) two thermal cycles of heating of the NFC (first stage of pellets extrusion and second stage of injection) can produce the degradation of fiber in case of high processing temperature, (c) injection molding is characterized by high level of shear stress in areas of the mold where the flow channel is quite thin, increasing the degradation level of the fiber, (d) the moisture content is more critical and the drying process of the compounding requires a better control, and (e) uneven distribution of fiber content along the injected part, due to difference in density and the dimensions of the fiber, which leads to the phase separation phenomenon [15].

In the bibliography, different natural fibers, even mixtures of them, have been tested by injection molding with significant range of dispersion in terms of mechanical properties. For instance, A.K. Bledzki et al. [16] tested the mechanical behavior of a composite of polypropylene (PP) with abaca, jute and flax (with different percentages of fiber, from 20 to 50%). Due to the typical problem of fiber–matrix compatibility, these authors used a coupling agent with maleic anhydride grafted with PP (MAH-g-PP), producing an improvement of mechanical properties, mainly the flexural, tensile and impact strength. The best Young’s modulus (5000 MPa) was obtained with Abaca fiber with PP/MAH-g-PP. Also, another research compared the behavior of different NFCs by injection molding with PP, using rice husks, wood flour, and sisal fibers [17], applying, as compatibilizer, MAH-g-PP. In this research, the sisal fiber composite performed better compared to the others, in terms of mechanical properties (about 4000 MPa of Young’s modulus and 49 MPa of maximum tensile stress for 30% of fiber content). A variant of flax fiber is the use of flax shives (500 µm) with PP/MAH-g-PP [18], with promising results (almost 90% of the Young’s modulus of flax fiber composite). The fiber treatment aims to improve the maximum strength, but in some researches the opposite effect was achieved. For example, in [19], the authors injected NFC based on PP and kenaf fiber, using different alkali treated and non-treated fiber contents (the percentage of NaOH was the same as the fiber content: 5, 10, and 15%, respectively). The results showed that the higher the non-treated fiber content, the higher the strength, while with the treated fiber the effect was the opposite (the higher the fiber and NaOH content, the lower the strength). Other authors have developed injected Nylon 6 NFC by using shells of coconut [20], confirming the good bonding of fiber–matrix when alkali treatment is applied. This NFC, with 15 wt.% of treated coconut shells, had an elastic modulus close to 3000 MPa and tensile strength over 75 MPa. Regarding the use of banana fiber in injected NFC, there are not reported works except those related to Abaca fiber, which is considered the most similar one to banana fiber (both are Musaceae and sometimes are confused in the bibliography). Most of the works of banana fiber focuses on the process of compression molding [12,21]. Either woven or nonwoven banana fiber has been developed to be used as reinforcement of composites by compression molding. Z. Ortega et al. [22] developed a fabric formed by yarn of 50 wt.% of banana fiber and 50 wt.% of wool. To manufacture the yarn, the banana fiber was enzymatically treated and mixed with other more flexible fiber (wool in this case, which led to worse mechanical properties) to facilitate the yarning process. The composite was produced by compression molding with 52 wt.% of textile and 48 wt.% of PP, without any coupling agent, giving as a result a Young’s modulus about 1600 MPa and maximum tensile stress of 26 MPa. A similar setup was applied to commercial textile of flax, obtaining a Young’s modulus and maximum stress of 2600 and 69 MPa, respectively. Another research with banana fiber composite by compression molding was produced by nonwoven of banana fiber (alkali treatment) and PP fiber [21]. The best results with NaOH treatment was 1860 MPa for the Young’s modulus and 40 MPa for the tensile strength.

The limitation of NFCs can be overcome by the hybridization of the composite by adding synthetic fiber (carbon, glass, etc.) with natural fibers, improving mechanical properties but keeping an acceptable damping factor [23].

The aim of the present work is to characterize the thermal and mechanical behavior of different thermoplastic composite polymers reinforced with short fiber of banana from the Canary Islands for its application in injection molding.

## 2. Materials and Methods

### 2.1. Fiber Extraction

**Figure 1:**
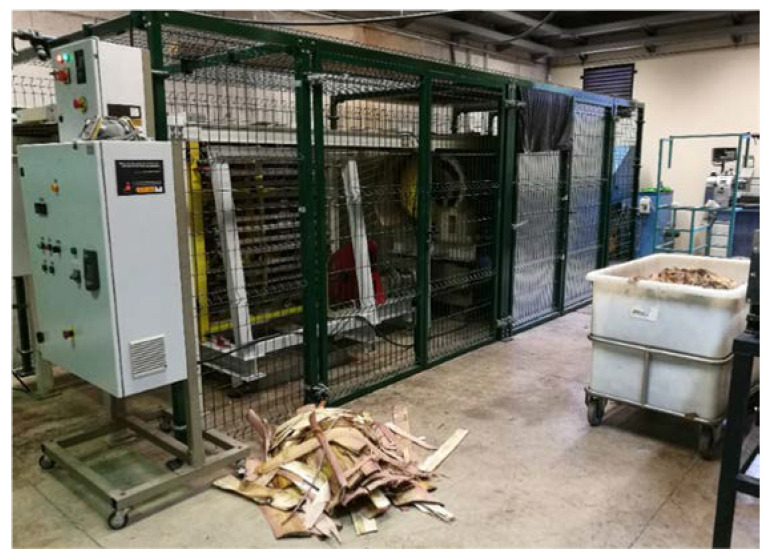
Pilot plant for extraction of banana fiber.

The banana fiber was extracted from the specie “Pequeña Enana”, grown in the Canary Islands (Spain). The pseudostems of the banana plant were supplied by farmers in the island of Gran Canaria. The leaves of the pseudostem were released from the core and processed in the pilot plant (Figure 1) developed by the University of Las Palmas de Gran Canaria [24]. This extracting system allows scraping the leave (length about 0.5 m), by linear and rotational movement, under an automatic process, obtaining clean banana fiber [25] with a global efficiency of 51% (percentage of obtained fiber compared to the total fiber available in the leaves).

**Figure 2:**
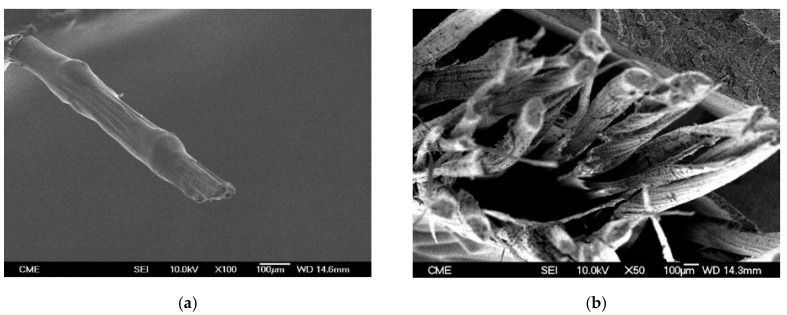
SEM images of: (**a**) a banana fiber and (**b**) banana fibers section.

Once the fiber is extracted, the next step is to chop the fiber in the following equipment of the pilot plant. The selected length of the chopped fiber was 2 mm, removing the longer fraction in a secondary process by rotational sieving. The banana fiber (Figure 2) has an average diameter of 0.180 mm [26]. The tensile modulus is 43 GPa and the tensile strength 891 MPa [3].

### 2.2. Treatment of Banana Fiber

**Figure 3:**
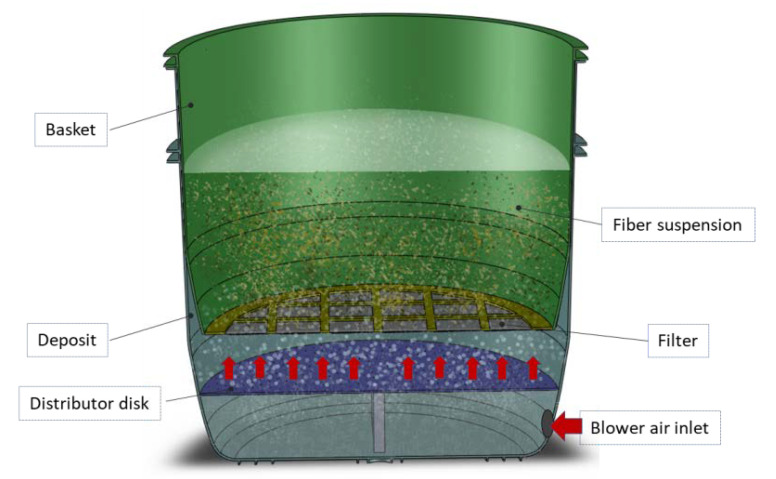
Bubble column reactor to treat the chopped fiber.

**Figure 4:**
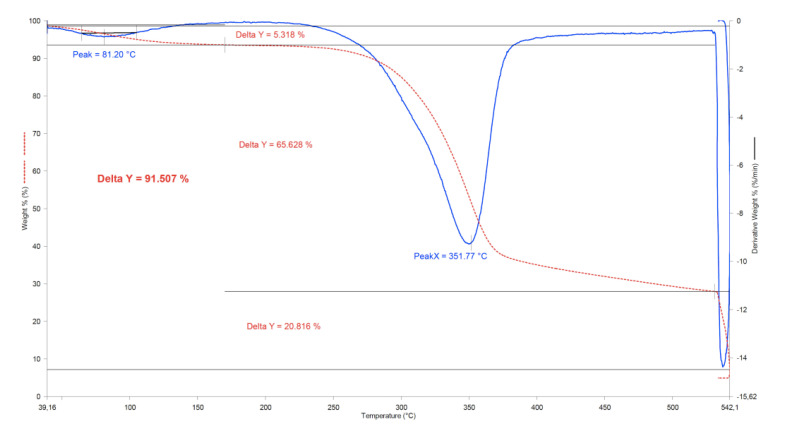
TGA and DTG of treated BF.

To improve the fiber and polymeric matrix integration, and to increase the degradation temperature of the banana fiber, an alkali treatment was applied [3,27]. A bubble column reactor with suspended solids (Figure 3) was designed and manufactured to produce batches of treated chopped fiber (Figure 4) with a solution of NaOH 1N. The capacity of the reactor is 200 L of NaOH 1N solution and a maximum amount of 5 kg of chopped fiber [27]. Two recirculating pumps and air agitation allow the treatment process, with the advantage that the NaOH solution can be used 8–9 times. To produce the treated fiber for the composite, batches of 2 kg of fiber were treated for 1 h at room temperature. The efficiency of the reactor in terms weight loss of fiber is about 80%, therefore from 1 kg of raw fiber it can be obtained 0.8 kg of treated fiber.

The wet treated fiber was centrifuged at 1000 rpm and dried in a continuous rotational drier by infrared lamps. After the drying process, the final moisture content of the treated fiber was approximately 12% (average value of more than 30 treated batches with three measurements of each one), ready for the following step of compounding extrusion with the thermoplastic matrix.

### 2.3. Characteristics of Treated Banana Fiber

The mass degraded in a Thermal Gravimetric Analysis (TGA) of the treated fiber (N_2_, heating rate of 5 °C/min) is shown in Table 2. The treated fiber presents a loss of mass (Table 2) that shows an improvement in terms of increasing the degradation temperature compared to untreated fiber [27]. This improvement allows heating the fiber during the compounding or injection molding process at higher temperature without degradation.

The mechanical properties of the treated fiber with NaOH 1N, in terms of tensile modulus and tensile strength, are 52 GPa and 687 MPa, respectively [3].

### 2.4. Polymeric Matrix

Several thermoplastics were used as polymeric matrix to obtain different combinations of banana fiber reinforced compounding. The commercial polymers used were ABS from LG HI121H-NP, high impact polystyrene (HIPS) from Synthos PS HI 552M and high density polyethylene (HDPE) from Egyptene HD 6070UA. The polymer matrix and percentage of banana fiber used are presented in Table 3. Different percentages were proposed according to the desired effect on the final functional parts to be manufactured with these composite materials. In the case of HDPE, the final application did not require as high mechanical properties as the final application of ABS and HIPS composites. For this reason, the amount of banana fiber was reduced by half.

### 2.5. Compounding Processes

All the compounds were obtained by extrusion through a LABTECH–LTE 20–40 twin screw extruder (Labtech Abtech Engineering Company LTD, Mueang Samut Prakan, Thailand). The default setup process consisted in a pressure up to 23 bar, a temperature distribution between 142 and 161 °C with 155 °C die temperature, 400 rpm screw speed and extrusion speed of 18.2 m/min. A pelletizer finalized the composite material with 4 mm length. 10 kg of compound were obtained for each polymeric matrix (ABS, HIPS, and HDPE) and each established BF percentage (Table 3).

Tensile test samples were injected in an Engel e-Max 440/100 machine (Engel Austria GmbH, Schwertberg, Austria), with a 40 mm screw diameter and maximum injection pressure of 1930 bar. A previous drying was applied at 100 °C for 24 h, before the material was stored in the dry production unit. The injection molding machine used was a KRAUSS MAFFEI, CX 50-180 (Krauss-Mafei-Wegmann GmbH, Munich, Germany), with a clamping force of 500 kN. As a reference, the main injection molding parameters of the ABS samples were as follows:

- Temperature on cylinder heating zones: 180, 190, 200, and 210 °C
- Plasticizing speed: 50 rpm
- Back pressure: 75 bar
- Switchover point: 6 mm
- Injection speed: 50 mm/s
- Injection pressure: 1000 bar
- Holding pressure and duration: 600 bar (10 s)
- Temperature of tempering unit: 50 °C (cooling time: 23 s)

### 2.6. Composite Characterization Test

#### 2.6.1. Thermal Gravimetric Analysis (TGA)

A TGA was carried out using a Perkin Elmer TGA 4000 device (PerkinElmer, Inc., Waltham, PA, USA) to analyze the thermal characteristics in specimens of approximately 10 mg and with two different atmospheres, nitrogen and oxygen, respectively. The initial nitrogen atmosphere allows degrading volatile material (organic components), while the final oxidizing conditions burns the carbonaceous material, leaving behind inorganic components (ash content). Before the measurement, the samples were prepared and weighted. Then, the thermal cycle was applied in nitrogen atmosphere, consisting in a tempering process for 1 min at 40 °C and a subsequent heating at a heating rate of 10 °C/min up to 550 °C. Next, the nitrogen atmosphere was changed to oxygen and the sample was heated at the same temperature (550 °C) for 10 additional minutes.

TGA was used to detect physical phenomena, such as volatilization of volatiles, the proportion of different components in the sample (organic, inorganic), and degradation. The first derivative of the TGA curve (derivative thermogravimetry, DTG) was also obtained as it provides more accurate information about the degradation temperature or the loss of mass at higher temperatures, since the decomposition at some temperature may not be complete when a new decomposition occurs at higher temperature.

#### 2.6.2. Differential Scanning Calorimetry (DSC)

Differential dynamic calorimetry measurements were achieved on a Mettler Toledo DSC 2 device (Mettler-Toledo International Inc., Columbus, OH, USA). The measurement was performed by heating and cooling the samples twice in a nitrogen atmosphere with a nitrogen flow rate of 20 mL/min. The temperature range of the measurements was from 0 to 180 ℃ and the heating rate was 10 °C/min.

The thermogram detects transitions as bands at different temperatures relevant to a given thermal transition. Thus, different thermal transitions can be determined, such as glass transition temperature (T_g_), melting temperature (T_m_), crystallization temperature (T_c_), melting enthalpy (ΔH_m_), crystallization enthalpy (ΔH_c_) and specific heat capacity (ΔC_p_).

#### 2.6.3. Melt Flow Index (MFI)

A LIYI, MFI LY-RR device (Dongguan Liyi Environmental Technology Co., Ltd., Dongguan, China) was used to perform MFI tests in accordance with ISO 1133:2020 [28]. For each polymer matrix, the mass and temperature used for the MFI test was selected according to the values used in the corresponding material datasheet (10 kg and 220 °C for ABS, 5 kg and 200 °C for HIPS and 180 °C and 2.16 kg for HDPE).

#### 2.6.4. Mechanical Tests

Tensile and flexural tests were performed on a Shimadzu AG–X plus 10 kN mechanical performance testing machine (Shimadzu Corporation, Kyoto, Japan).

For each composite combination, 5 samples were produced according to the standard ISO 527-2:2012 [29] for conducting the tensile test and determining the elastic modulus, tensile strength and elongation at break. The dimensions of test specimens were the ones specified by de referenced ISO standard. The bone sample was then inserted between the jaws 50 mm apart. The test was run at a load speed of 1 mm/min up to 0.25% elongation, and then at a speed of 50 mm/min up to break.

In the bending test, 5 samples were also tested in accordance with ISO 178:2012 [30]. The displacement rate of the upper point was 2 mm/min and the distance between the supports was 64 mm.

The impact tests were performed on a Charpy LY-XJJDS device (Dongguan Liyi Environmental Technology Co., Ltd., Dongguan, China) in accordance with ISO 179-1:2010 [31]. For both impact strength and notch impact strength, 10 samples with a thickness of 2 mm and a width of 5 mm were used. The distance between the supports was 60 mm. A hammer of 2 J was used to measure the impact toughness of the matrices and composites with 10% and 20% of banana fibers. For the composites with 30% banana fibers and for the notch impact toughness tests 1 J hammer was used.

#### 2.6.5. Dynamic Mechanical Analysis (DMA)

A DMA was applied to measure the influence of temperature on the mechanical properties of the composites developed. A Perkin Elmer DMA 8000 device (PerkinElmer, Inc., Waltham, PA, USA) was used for this purpose. Samples with 5 mm width, 3 mm thickness and 12 mm length were clamped in a double clamp. The frequency and amplitude were set to 1 Hz and 0.02 mm respectively. Samples were heated from 25 to 150 °C with a heating rate of 2 °C/min.

In this case, the following tensile properties were determined: storage E modulus (E′), loss modulus (E″) and loss factor (tan δ). The storage E module (E′) represents the elastic response of the material under mechanical loading, which can be restored to its original state. The loss modulus (E″) represents the part of rigidity where the mechanical load is converted to internal energy by friction causing plastic deformation. The damping factor (tan δ) represents the ratio of the loss modulus to the storage E module.

## 3. Results and Discussion

### 3.1. Thermal Characterization

**Figure 5:**
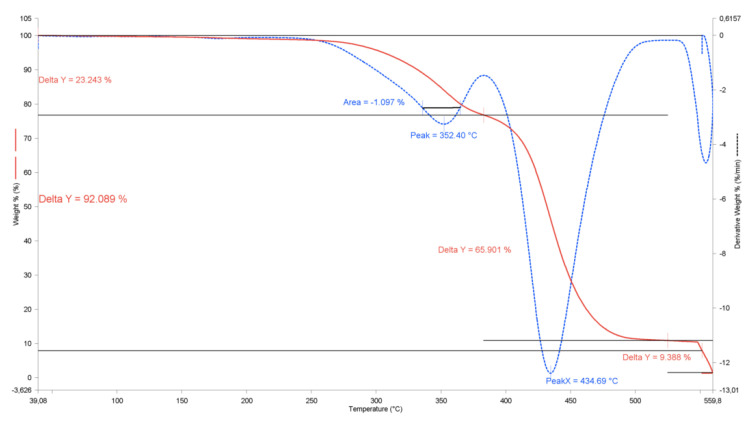
TGA and DTG of ABS + 30% BF composite.

Table 4 shows the thermal stability of the compounding samples with BF decomposition as a percentage of weight loss and the temperature peaks of BF and polymeric matrix as T_1_ and T_2_, respectively. The degradation temperature of BF was similar among the different samples, with peaks between 344.1 °C and 358.0 °C, indicating that the thermal behavior of BF is homogeneous and in accordance with the typical range of degradation peaks for alkali treated BF [3]. This allows the use of BF composites for most of the applications of composite materials without a noticeable loss of the thermal properties required for the specific application. On the other hand, the decomposition peaks of the polymeric matrices were also similar within each polymer group, without relevant difference related to the percentage of BF used, although it was found that these peaks were higher in the ABS and HIPS composites compared with the pure polymer. This behavior is in line with the results observed by Reddy et al. [32], due to the interaction and synergies of the fiber–matrix that increase the degradation temperatures. However, HDPE-based compounds showed no change in degradation temperatures, explained by the minimal gas permeation barrier between fiber and polymer [33]. As expected, the mass loss of BF increased with the BF percentage used in the composite, but it was less pronounced at higher BF content. The last column shows the ash content, which also increased with the BF percentage. The TGAs of the banana fiber (Figure 4) showed ash values of approximately 21%, while the pure polymer TGAs showed a minimum ash content (0.2–0.6%). Therefore, BF introduces these ash contents (up to 9.4%, Figure 5) with some irregularity due to uneven mixtures of the BF in the compounds.

The DSC analyzes showed a slight decrease in the glass transition temperature with the increase of BF content, as well as a gradual decrease in the specific heat capacity in both ABS and HIPS composites (Table 5). In ABS, the melting temperature was not significantly modified, with a variation of less than −0.5% for the highest concentration of BF with respect to the pure matrix, and an unimportant decrease in the crystallization temperature. Therefore, the properties of phase transition of this compound remained unchanged, at least in terms of temperature. However, a decrease in the specific heat was observed with the increase of BF content, which may lead to an advantage for thermal conformations such as injection molding.

On the other hand, the melt enthalpy ranged between 1.2 and 1.8 J/g, probably due to previous heat treatments that influence the crystallization and thermal behavior. The specific crystallization enthalpy decreased to a greater extent as the BF content was increased. A reduction of up to 38.89% was observed for 30% BF, decreasing from 1.8 J/g (matrix) to 1.1 J/g, which indicates a decrease in crystallization in the compounds with greater BF reinforcement. Other authors have observed this same effect, concluding that fibers hinder the movement of the molecular chains of the polymers, thereby reducing crystallinity [34]. However, in other studies [35], natural fibers produced an increase in crystal growth, with BF acting as nucleating agents. This opposite effect on crystallinity may be attributed to several influential factors such as different heating rates and materials used. Furthermore, a smaller decrease was also observed in the specific enthalpy of fusion, although the variability of the results in this case requires a more exhaustive study to analyze the effects on the thermal behavior in the fusion phase.

**Figure 6:**
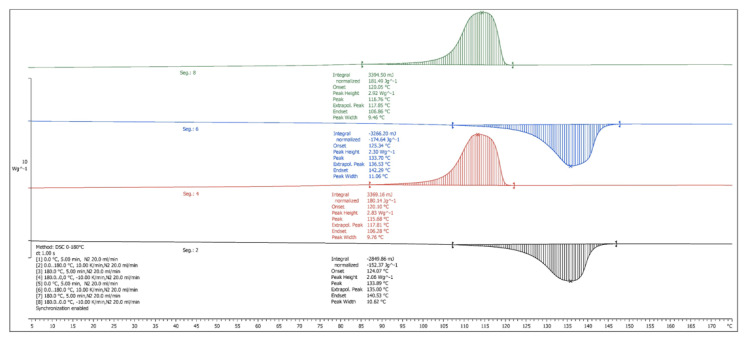
DSC curves of high density polyethylene (HDPE) + 15% BF composite.

In the case of HDPE, more pronounced effects were observed compared with ABS. A large decrease in the specific fusion enthalpy (from 183.5 to 152.4 J/g) took place for the compound of 15% of BF (Figure 6), indicating a great reduction in crystalline components. This trend was also registered in the cooling crystalline transformation, with a reduction of the specific cooling enthalpy as the BF content increased, from 205.0 J/g for pure HDPE to 180.1 J/g for a compounding with 15% BF. The melting and crystallization temperatures remained however unchanged.

**Figure 7:**
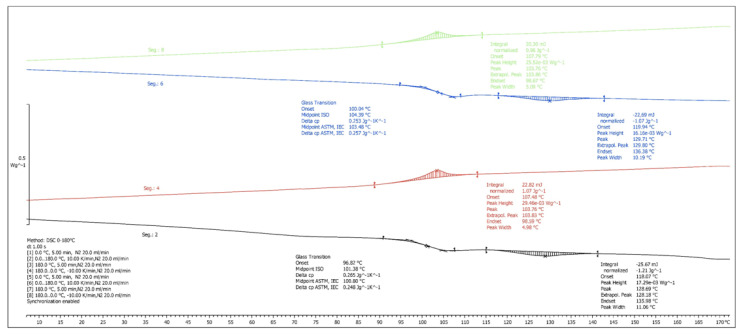
DSC curves of ABS + 30% BF composite.

Table 6 shows the effects of the second scan cycle, matching the thermal history of all the samples. The results reflect a trend very similar to the first cycle, with a minimal variation in temperatures but with significant variations in the specific enthalpies of fusion and crystallization, especially for the samples with the highest percentage of BF. Both ABS + 30% BF (Figure 7) and HDPE + 15% BF (Figure 6) maintained a reduction of the fusion energy of 15.3% and 15.9%, respectively, with respect to the base materials, as well as a decrease in the energy released in the cooling phase of 37.5% and 13.2% respectively, fundamentally producing a significant decrease in crystal growth in the ABS samples. The samples with lower BF contents also showed enthalpy reductions in all cases, which is in line with the results obtained for other PP and polyethylene (PE) composites reinforced with natural fibers [36,37]. However, as mentioned before, the fiber may hinder the migration and diffusion of polymer molecular chains (thus reducing crystallization), or, in some specific cases (long fiber or controlled shear stress during injection), may have a nucleating effect (increase of crystallization). Therefore, the effect of the inclusion of natural fiber on crystallinity depends on the type of polymer used and heating cycles applied.

The effects produced by the incorporation of the reinforcements on the melt flow index are evident, as shown in Table 7. A considerable loss of fluidity was observed, mainly due to the obstruction that the fiber produces on the mobility of the molecular chains, reaching reductions of over 50% in the composites with the highest BF content, and being especially critical in the case of HIPS. This behavior could also indicate variations in the size of the matrix molecular structure due to the breakdown of the molecular chains and, therefore, to the decrease in molecular weight [38].

### 3.2. Mechanical Properties

**Figure 8:**
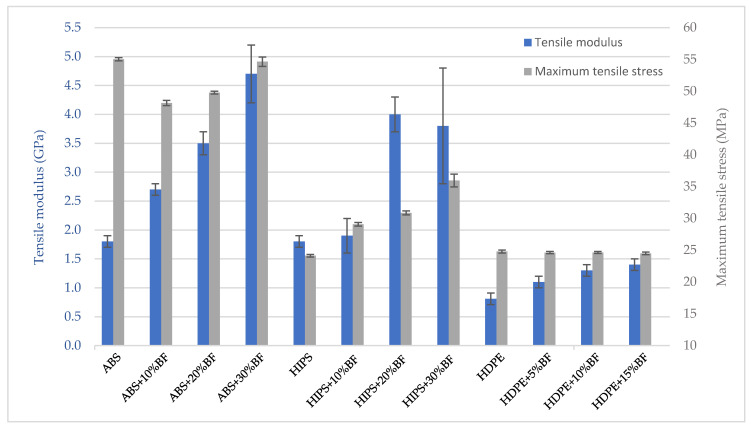
Tensile elastic modulus and maximum tensile stress of composites.

Figure 8 shows the results of the tensile mechanical tests, which are quite similar for HDPE and ABS composites. As it can be observed, the tensile modulus increased progressively with the percentage of fiber for all the polymer matrices. The greatest increase in stiffness of HDPE reached 72.8% compared to the pure matrix, for a fiber content of 15%, reaching a tensile modulus of 1.4 GPa. Despite the greater rigidity of ABS, the increases of the mechanical parameters in this material were higher, practically doubling the tensile modulus in the composite with 20% BF (3.5 GPa) and reaching maximum values of 4.7 GPa for the highest concentration of BF. In the HIPS specimens, it was possible to double the values of the tensile modules except for the 10% BF samples.

As for the maximum tensile stress, the behavior of the compounds was heterogeneous. In HDPE composites remained practically unchanged, in ABS slightly decreased for medium loads and in HIPS increased in all cases.

The same trends in terms of stiffness were observed in the flexural tests (Table 8), with an increase in the flexural modulus with the BF content in all the cases, in parallel with the decrease in the maximum elongation at break. However, the results in terms of maximum flexural stress were better compared to the tensile ones, since the increases in maximum stress with the BF content were significant for all the polymer matrices except for ABS, in which it practically remained unchanged. Therefore, the fiber performed better under flexural loads than under tensile conditions.

The different behavior of the composites (in terms of maximum tensile stress between the polymeric matrices) is mainly due to the following factors: (a) different efficiency of alkaline treatment and behavior of the matrices in terms of fiber–matrix coupling and (b) inhomogeneous distribution of the fiber into the polymeric matrix, during the injection process, because of the formation of fiber bundles, the level of viscosity of each polymer and the biological origin of the fiber.

To observe the distribution, clustering and fiber orientation, samples of each composite were sliced with a uniform thickness of 1.5 mm and several images were taken in an optical microscope.

**Figure 9:**
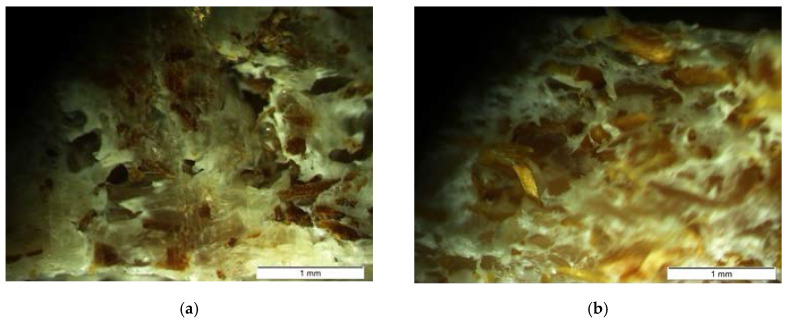
(**a**) Broken sample section from ABS 10% BF 5× and (**b**) broken sample section from HDPE 15% BF 5×.

Regarding the fiber–matrix interaction, the consequences of higher tensile strength of the fiber with respect to the matrix can be appreciated in the sections of rupture. In the ABS samples (Figure 9a), the fiber is separated from the matrix, producing visible voids and intact bare fibers, which may indicate a lower matrix–fiber adhesion. In the HDPE samples (Figure 9b) and also the HIPS ones (not shown), there are no clear fiber gaps but rather fraying of the matrices, which can be understood as a better fiber–matrix performance. This explains the improvement of the maximum flexural stress in these two composites (Table 8).

In contrast to synthetic fibers, banana fibers have uneven geometric characteristics and defects that are generated during the growth of the plant, such as nodes, sliding planes, twisted bands, or dislocations (Figure 2b). Moreover, the biological origin of the fiber leads to a non-constant transverse dimension (Figure 2a), being the areas closer to the root thicker than those farther away. For this reason, it is important to standardize the particle size of the plant fibers for its use as reinforcement.

As for the forming processes, extrusion and injection molding produce higher fiber attrition and breakage [39,40], generating fibers with variable lengths and diameters and mainly affecting the shear stress, the residence times and thermal fields. As a consequence of the variable size and usually low concentrations of the fiber, the risk of an inefficient fiber–matrix packaging is higher, and therefore its mechanical performance may be reduced.

**Figure 10:**
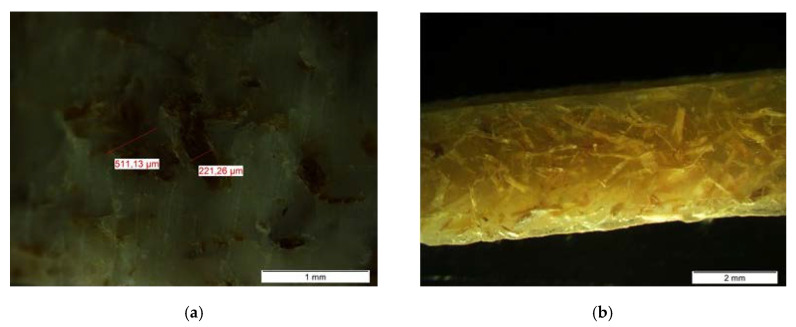
(**a**) Broken sample section from high impact polystyrene (HIPS) 10% BF 5× and (**b**) Sample of HDPE 10% BF 2×.

Figure 10a shows clusters of fibers in different areas of the injected pieces, (uneven fiber distribution). Figure 10b shows the variability of the orientation of the fibers, which are not necessarily aligned with the orientation of the flow. Fibers with non-uniform thicknesses and lengths can also be observed, as well as breakages and fraying, causing changes in the behavior of composites.

As expected, the increase in BF significantly influenced the storage modulus of elasticity, considerably increasing its value in the highest BF contents, where increases of 34.6% were observed for ABS, 72.2% in HIPS, and 58.3% in HDPE (Table 9), produced by the greater transfer of stress from the matrix to the fibers. The increase in the glass transition temperature is clearly reflected in the DMA tests where the mobility of the matrix molecules is more restricted with the increase in the BF content. The increase in fiber content produces a gradual decrease in the loss factor, thus producing a material with less damping capacity and greater stiffness.

**Figure 11:**
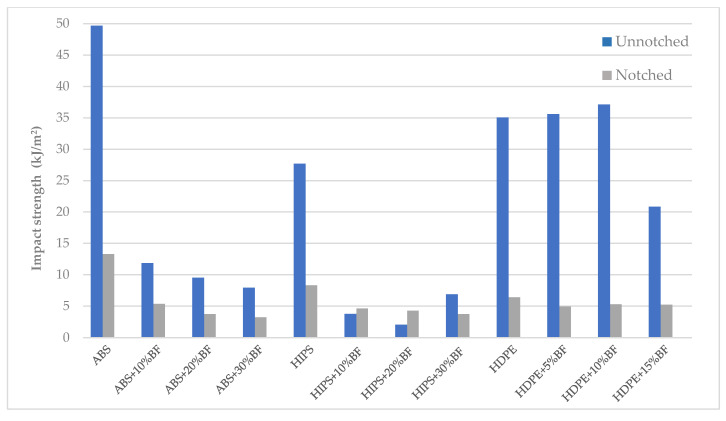
Impact strength of composites (notched and unnotched).

Regarding the impact tests (Figure 11), the inclusion of BF led to lower impact resistance due to a greater stiffness introduced by the fiber, decreasing energy absorption.

## 4. Conclusions

This work presents the thermal and mechanical characterization of thermoplastic composite polymers reinforced with banana fiber. ABS, HIPS, and HDPE were used as matrices, reinforced with different percentages of banana fiber to analyze their behavior in injection molding processes. The different thermal tests carried out showed a thermal behavior of the composites similar to the matrices, without great changes in the different thermal transitions, although with an expected reduction in the MFI. Neither was a worsening in the degradability observed. Therefore, from a thermal point of view, the use of banana fiber as reinforcement in composites for its conformation through injection molding processes is viable.

As for the mechanical characteristics, the composites showed in all cases an increase in the elastic modulus with the increase of banana fiber content, indicating that reinforcement allows more rigid materials. This increase in stiffness was also confirmed with the impact tests, where a decrease in the energy absorption was observed with the increase of banana fiber content. On the other hand, the maximum stresses remained practically unchanged in the tensile test (except in HIPS composites), while in the flexural tests were higher with the increase of banana fiber content (except for the ABS matrix composites). These results show some variability in the fiber–matrix interaction depending on the type of matrix used, being more efficient in HIPS and HDPE than in ABS.

Therefore, the use of banana fiber can be especially promising as a natural reinforcement in the production of parts subjected to bending loads, which is one of the most common load cases. However, it is important to note that the effects produced by the reinforcement can vary depending on the matrix used, and especially in the quality and uniformity of the fiber used.

## Figures and Tables

**Table 1:** Mechanical data of several fibers.

Fiber	Specific Tensile Strength (MPa·cm^3^/g)	Specific Elastic Modulus (MPa·cm^3^/g)	Reference
Abaca	540.77	19.23	[6]
Cotton	190.07–500.00	3.64–7.88	[7,8]
Bamboo	358.33–239.56	18–32.97	[7,8]
Cane	142.86–285.71	75.51	[9]
Hemp	210–510	20–46.67	[7,8,10]
Ceiba	300	12.9	[7]
Coco	104.35–827.59	3.48–131.03	[8,9]
Kenaf	2184.62–7005.88	161.54–352.94	[7,8]
Flax	230.00–1000.00	15.93–48	[7,8]
Palm (oil)	101.29–160.00	9.57–9.03	[8]
Palm (from dates)	269.98–431.97	151.19	[9]
Pineapple	287–1130	34.17–57	[7,8,10]
Banana tree	240.00–677.04	30.67	[7,8,9,10]
Canary banana tree	793.08	35.38	[6]
Ramie	400.00–605.16	15–82.58	[7,8]
Rosella	226.67–437.50	21.25–22.67	[8]
Sisal	248.23–441.38	9.08–21.43	[7,8,9,10]
Jute	140–537.93	10–39	[7,8]
Fiberglass	984.25	22.05–28.35	[7,8,9,10]

**Table 2:** Mass degraded (%) in Thermal Gravimetric Analysis (TGA) analysis [3].

	105–220 °C	220–290 °C	290–330 °C	330–470 °C	470–900 °C
Untreated fiber	3.7%	15.4%	33.1%	15.2%	12.9%
Treated fiber	2.4%	10.8%	24.4%	33.7%	11.2%

**Table 3:** Polymer matrix and percentage of banana fiber (BF).

Matrix	wt.% Banana Fiber			
ABS	0 ^1^	10	20	30
HIPS	0 ^1^	10	20	30
HDPE	0 ^1^	5	10	15

^1^ Pure polymer.

**Table 4:** TGA of BF composites.

Sample	Weight Loss ^2^ (%)	Peak T_1_ (°C)	Peak T_2_ (°C)	Ashes (%)
ABS ^1^	-	-	429.8	0.6
ABS + 10% BF	10.9	354.7	437.2	3.1
ABS + 20% BF	15.7	353.8	435.1	3.4
ABS + 30% BF	23.2	352.4	434.7	9.4
				
HIPS ^1^	-	-	441.7	0.4
HIPS + 10% BF	11.2	358.0	446.0	2.9
HIPS + 20% BF	17.7	357.7	447.1	3.8
HIPS + 30% BF	23.2	357.9	448.8	4.2
				
HDPE ^1^	-	-	490.3	0.2
HDPE + 5% BF	4.1	348.6	489.9	0.7
HDPE + 10% BF	7.6	344.1	490.2	0.6
HDPE + 15% BF	9.2	354.2	488.1	2.6

^1^ Pure polymer. ^2^ BF decomposition.

**Table 5:** First cycle of Differential Scanning Calorimetry (DSC) for BF composites.

Sample	T_g_ (°C)	ΔC_p_ (J/g)	T_m_ (°C)	ΔH_m_ (J/g)	T_c_ (°C)	ΔH_c_ (J/g)
ABS	102.1	0.3	129.3	1.5	104.4	1.8
ABS + 10% BF	102.1	0.3	129.4	1.3	104.1	1.4
ABS + 20% BF	101.7	0.2	128.5	1.8	103.9	1.1
ABS + 30% BF	100.8	0.2	128.7	1.2	103.8	1.1
						
HIPS	95.1	0.4	-	-	-	-
HIPS + 10% BF	94.1	0.3	-	-	-	-
HIPS + 20% BF	94.0	0.2	-	-	-	-
HIPS + 30% BF	93.3	0.2	-	-	-	-
						
HDPE	-	-	134.1	183.5	116.7	205.0
HDPE + 5% BF	-	-	134.3	166.8	117.3	193.0
HDPE + 10% BF	-	-	133.0	155.2	116.4	182.9
HDPE + 15% BF	-	-	133.9	152.4	115.7	180.1

**Table 6:** Second cycle of DSC for BF composites.

Sample	T_g_ (°C)	ΔC_p_ (J/g)	T_m_ (°C)	ΔH_m_ (J/g)	T_c_ (°C)	ΔH_c_ (J/g)
ABS	103.6	0.3	135.0	1.3	103.9	1.6
ABS + 10% BF	103.5	0.3	129.8	1.4	104.1	1.1
ABS + 20% BF	103.1	0.2	130.3	1.2	104.5	1.0
ABS + 30% BF	103.5	0.3	129.7	1.1	103.8	1.0
						
HIPS	93.3	0.2	-	-	-	-
HIPS + 10% BF	93.3	0.2	-	-	-	-
HIPS + 20% BF	93.1	0.2	-	-	-	-
HIPS + 30% BF	92.5	0.2	-	-	-	-
						
HDPE	-	-	133.2	207.6	117.9	209.1
HDPE + 5% BF	-	-	133.4	193.0	117.9	193.0
HDPE + 10% BF	-	-	133.6	183.3	118.1	186.8
HDPE + 15% BF	-	-	133.7	174.6	116.8	181.5

**Table 7:** Results of Melt Flow Index (MFI) test.

Sample	T (℃)	m (kg)	MFI (g/10 min)
ABS	220	10.00	6.4
ABS + 10% BF	220	10.00	4.6
ABS + 20% BF	220	10.00	3.2
ABS + 30% BF	220	10.00	1.8
			
HIPS	200	5.00	10.8
HIPS + 10% BF	200	5.00	6.2
HIPS + 20% BF	200	5.00	2.9
HIPS + 30% BF	200	5.00	0.9
			
HDPE	180	2.16	6.8
HDPE + 5% BF	180	2.16	5.4
HDPE + 10% BF	180	2.16	4.5
HDPE + 15% BF	180	2.16	3.2

**Table 8:** Flexural test results.

Sample	Flexural Modulus (GPa)	Maximum Stress (MPa)	Maximum Elongation (%)
ABS	2.4 ± 0.0	76.7 ± 0.5	4.8 ± 0.3
ABS + 10% BF	3.4 ± 0.0	75.4 ± 0.4	3.6 ± 0.1
ABS + 20% BF	4.2 ± 0.1	76.3 ± 1.1	2.5 ± 0.1
ABS + 30% BF	5.2 ± 0.0	77.3 ± 1.4	1.9 ± 0.5
			
HIPS	1.7 ± 0.0	30.6 ± 0.3	5.8 ± 0.5
HIPS + 10% BF	3.1 ± 0.1	45.3 ± 0.5	2.9 ± 0.4
HIPS + 20% BF	3.2 ± 0.4	44.3 ± 0.3	2.3 ± 0.4
HIPS + 30% BF	4.7 ± 0.1	48.5 ± 1.1	1.5 ± 0.1
			
HDPE	1.0 ± 0.0	22.9 ± 0.1	6.9 ± 0.1
HDPE + 5% BF	1.1 ± 0.0	24.2 ± 0.1	6.6 ± 0.1
HDPE + 10% BF	1.4 ± 0.0	26.9 ± 0.1	6.3 ± 0.1
HDPE + 15% BF	1.8 ± 0.0	29.6 ± 0.1	5.8 ± 0.1

**Table 9:** Dynamic Mechanical Analysis (DMA) of BF composites.

Sample	E′ (GPa, 30 °C)	tan ∂	T_g_ (°C)
ABS	2.6	1.7	114.1
ABS + 10% BF	2.5	1.6	116.2
ABS + 20% BF	3.1	1.2	117.2
ABS + 30% BF	3.5	1.0	117.8
			
HIPS	1.8	1.6	107.1
HIPS + 10% BF	2.1	1.5	108.7
HIPS + 20% BF	2.5	1.3	110.1
HIPS + 30% BF	3.1	1.2	110.6
			
HDPE	1.2	-	-
HDPE + 5% BF	1.4	-	-
HDPE + 10% BF	1.6	-	-
HDPE + 15% BF	1.9	-	-
